# Supplementary material for: Parental-effect gene-drive elements under partial selfing, or why do Caenorhabditis genomes have hyperdivergent regions?
Source: Genetics. 2024 Oct 30;229(1):iyae175. doi: 10.1093/genetics/iyae175 (PMC11708918; doi:10.1093/genetics/iyae175)
Supplement: iyae175_Supplementary_Data [file iyae175_supplementary_data.zip › File_S1_GENETICS-2024-307305.pdf]

Special Case 1: a single *Medea*

Wade and Beeman (WADE AND BEEMAN 1994) showed that a *Medea* element will invade and sweep to fixation in a randomly mating population. That result holds under partial selfing.

With $k_2 = 0$, so that only allele M_1 has *Medea* activity, the recursion equation for M_1 frequency p (eq. 3) becomes $p' = \frac{p}{\bar{w}}$, with $\bar{w} = 1 - \frac{Yk_1}{2}\left(\frac{S}{2} + (1-S)q\right)$. Because $\bar{w}$ is always less than or equal to 1, the change in M_1 allele frequency (p'-$p = \Delta p$) is always positive (when $Y > 0$) or 0 (when $Y = 0$). Whenever $S<1$ and $0<p<1$, outcrossing will generate heterozygotes, keeping Δp positive and driving M_1 to fixation.

When rare, M_1 alleles increase in frequency most quickly under high rates of outcrossing (Figure S1). This is due both to the higher rate of production of heterozygotes and to the high probability that outcrossing heterozygotes will mate with the prevalent M_1-susceptible M_2 homozygotes, inducing the selective deaths of $k_1/2$ of the cross progeny. When the M_1 allele becomes very common, however, outcrossing is less efficient, as heterozygotes will tend to mate with the now-common M_1 homozygotes, resulting in 100% viability of the cross. If a heterozygote instead reproduces by self-fertilization, it will reliably generate and kill off $k_1/4$ of its progeny. The result is that partial selfing actually hastens the fixation of the *Medea* allele (Figure S1). This result was previously shown by stochastic simulation (NOBLE *et al.* 2021).

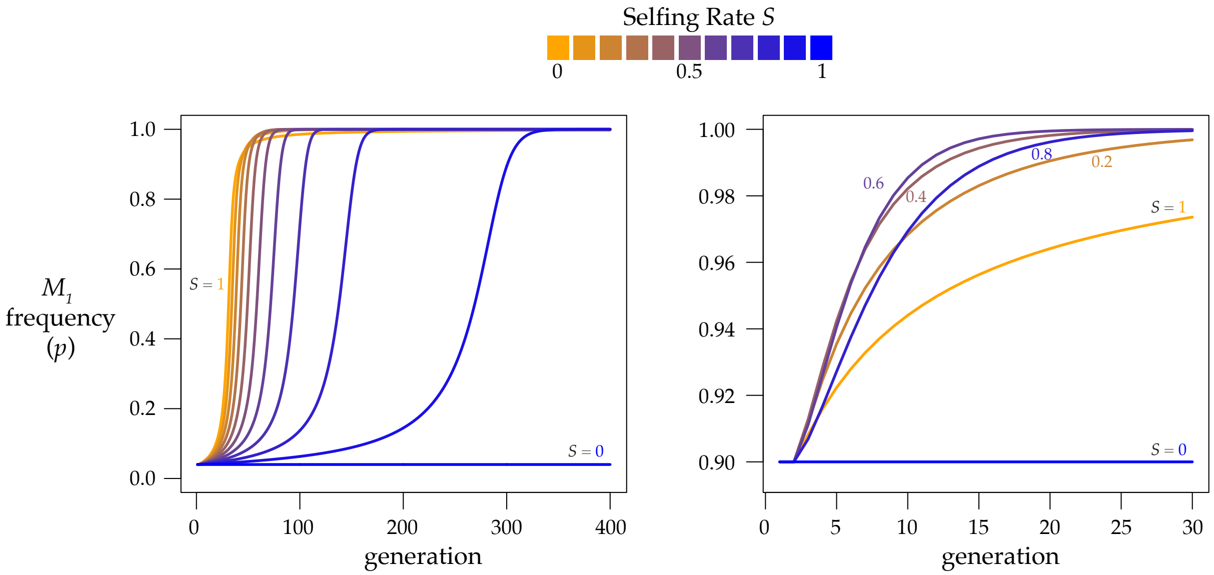

Figure S1. With outcrossing or partial selfing, a *Medea* element sweeps to fixation. These plots show results found by iterating equation 2 for different values of selfing rate S from 0 to 1, with penetrance $k_1 = 1$. At left, with initial allele frequency $p = 0.04$, selfing slows the spread of the *Medea*. At right, with initial allele frequency $p = 0.90$, partial selfing hastens the fixation of the *Medea*.

Special Case 2: Equal-penetrance antagonistic *Medea* alleles

When the ratio of penetrances $r = 1$ (i.e., $k_1 = k_2 = k$), $\bar{w}$ is simply 1-$Yk/2$ and the recursion for M_1 frequency p works out to $p' = S\frac{p-\frac{Yk}{4}}{\bar{w}} + (1-S)p$. The second term shows that under random mating (S=0), allele frequencies do not change; $p' = p$ for all p. This bizarre result, that alleles are neutral despite very strong selection at the genotypic level and dramatically reduced population mean fitness, was initially discovered by Hedrick (1997), who was analyzing an instance of maternal-fetal interaction analogous to *Medea* elements (WADE 2000). That this phenomenon yields genetic drift in finite populations was shown by stochastic simulation (NOBLE *et al.* 2021).

The difference equation is $\Delta p = S\left(\frac{Yk}{4-2Yk}\right)(2p-1)$. Recalling that $p = 0$ and $p = 1$ are equilibria, we find an additional equilibrium at $\hat{p} = 0.5$. This equilibrium is unstable under partial selfing. When $0>S>1$ and $0<p<0.5$, Δp is negative and the rarer allele is eliminated as p goes to 0. When M_1 is the common allele, $0.5>p>1$, Δp is positive and again the rarer allele is eliminated as p goes to 1. This pattern of positive frequency dependence was previously discovered by Wade (2000), who used a model with a fixed inbreeding parameter F to extend Hedrick's (1997) results.

The dynamics are complicated (Figure S2), as they depend on interactions among S, k, Y, and p. In general, the rarer allele is eliminated most rapidly at intermediate selfing rates, while both high and low rates of selfing result in a slower elimination of the rarer allele.

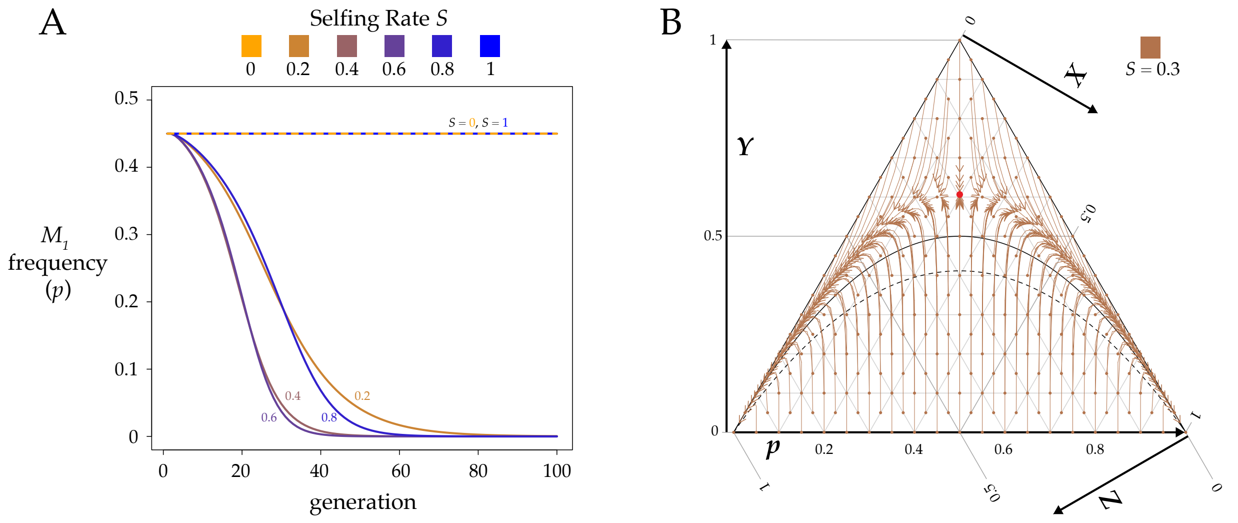

Figure S2. Partial selfing causes the elimination of the rarer of two equally penetrant *Medeas*. **A.** This plot shows allele frequencies found by iterating equation 2 for 6 values of S from 0 to 1, with $k = 0.9$ and starting from allele frequency $p = 0.45$ and heterozygosity $Y = 0$. The dashed orange and blue line represents results for both S=0 and S=1. **B.** This ternary (De Finetti) plot shows evolution of this system ($k = 0.9$, $S = 0.3$) in genotype frequency space (equation 1). Each segment starts from a small dot, a combination of the three genotype frequencies (points spaced every 0.05). Each segment then plots genotype frequencies through six generations, with the arrows pointing to the position of the population in generation 6. The solid black curve shows genotype frequencies at Hardy-Weinberg equilibrium, and the dashed curve shows genotype frequencies under the neutral extended Hardy Weinberg equilibrium with selfing. The red dot shows the unstable internal equilibrium at $\hat{p} = 0.5$ and $\hat{Y}$ given by equation 6 (approximately 0.61 for these parameter values). The stable equilibria are in the lower corners, at Z=1 (M_2 fixed) and X=1 (M_1 fixed).

Equilibrium heterozygosity under obligate outcrossing for this equal penetrance case was found by Hedrick (HEDRICK 1997). The general version with selfing is given by

$$\hat{Y} = \frac{1-\frac{S}{2}-\sqrt{\frac{S^2}{4}+1-S+(S-1)k}}{k} \text{ at } \hat{p} = 0.5.$$

As shown in figure S2B, heterozygosity is elevated considerably above the value expected under neutrality ($2pq(1-\hat{F}_0)$, where the equilibrium neutral fixation index $\hat{F}_0$ is $S/(2\text{-}S)$). The elevation is greater for high k and low S. The fixation index ($F = 1 - \hat{Y}/2pq$) is actually negative for a large part of the parameter space (at p=0.5, F<0 when $S < k/2$). In other words, there is an absolute excess of heterozygotes, even in the face of selfing.

Recursions for *Medea* elements under *Caenorhabditis*-type androdioecy

Taking into account the chromosomal sex determination mechanism in *Caenorhabditis*, each selfing results in hermaphrodite progeny only (XX), while each outcross results in 50% hermaphrodite, 50% male progeny. An outcross results in *b* hermaphrodite progeny for every 1 hermaphrodite that arises from a selfing; *b* is 0.5 if a cross results in the same number of progeny as a selfing, while it can be greater than 0.5 if a cross yields more progeny than a selfing.

Tracking the frequencies of different matings and the genotypes of their progeny, we can write *Medea* recursions in *X, Y, Z*, with *h* and *m* subscripts to specify hermaphrodite and male values respectively:

Egg-parent	Sperm-parent	Frequency	Surviving Progeny Genotypes: Hermaphrodites			Males		
			M_1M_1	M_1M_2	M_2M_2	M_1M_1	M_1M_2	M_2M_2
M_1M_1		SX_h	1					
M_1M_2		SY_h	$(1-k_2)/4$	$1/2$	$(1-k_1)/4$			
M_2M_2		SZ_h			1			
M_1M_1	M_1M_1	$(1-S)X_hX_m$	b			b		
M_1M_1	M_1M_2	$(1-S)X_hY_m$	$b/2$	$b/2$		$b/2$	$b/2$	
M_1M_1	M_2M_2	$(1-S)X_hZ_m$		b			b	
M_1M_2	M_1M_1	$(1-S)Y_hX_m$	$b(1-k_2)/2$	$b/2$		$b(1-k_2)/2$	$b/2$	
M_1M_2	M_1M_2	$(1-S)Y_hY_m$	$b(1-k_2)/4$	$b/2$	$b(1-k_1)/4$	$b(1-k_2)/4$	$b/2$	$b(1-k_1)/4$
M_1M_2	M_2M_2	$(1-S)Y_hZ_m$		$b/2$	$b(1-k_1)/2$	$b/2$	$b(1-k_1)/2$	
M_2M_2	M_1M_1	$(1-S)Z_hX_m$		b			b	
M_2M_2	M_1M_2	$(1-S)Z_hY_m$		$b/2$	$b/2$		$b/2$	$b/2$
M_2M_2	M_2M_2	$(1-S)Z_hZ_m$			b			b

The recursions for hermaphrodites:

$$X'_h = (S\left(X_h + \left(\frac{1-k_2}{4}\right)Y_h\right) + b(1-S)\left(X_hX_m + \frac{X_hY_m}{2} + \left(\frac{1-k_2}{2}\right)Y_hX_m + \left(\frac{1-k_2}{4}\right)Y_hY_m\right))/\overline{w}_h$$

$$Y'_h = (S\frac{Y_h}{2} + b(1-S)\left(X_hZ_m + \frac{Y_h+Y_m - Y_hY_m}{2} + Z_hX_m\right))/\overline{w}_h$$

$$Z'_h = (S\left(Z_h + \left(\frac{1-k_1}{4}\right)Y_h\right) + b(1-S)\left(Z_hZ_m + \frac{Z_hY_m}{2} + \left(\frac{1-k_1}{2}\right)Y_hZ_m + \left(\frac{1-k_1}{4}\right)Y_hY_m\right))/\overline{w}_h$$

$$\overline{w}_h = S\left(1 - Y_h\left(\frac{k_1 + k_2}{4}\right)\right) + b(1-S)\left(1 - \frac{Y_h}{2}\left(X_mk_2 + Y_m\left(\frac{k_1+k_2}{2}\right) + Z_mk_1\right)\right)$$

The recursions for males are simply the outcrossing part of the numerators above, normalized to the male-specific $\overline{w}_m$, which is the outcrossing part of the denominator.

$$X'_m = \left(X_h X_m + \frac{X_h Y_m}{2} + \left(\frac{1-k_2}{2}\right) Y_h X_m + \left(\frac{1-k_2}{4}\right) Y_h Y_m\right))/\overline{w}_m$$
$$Y'_m = \left(X_h Z_m + \frac{Y_h + Y_m - Y_h Y_m}{2} + Z_h X_m\right)/\overline{w}_m$$
$$Z'_m = \left(Z_h Z_m + \frac{Z_h Y_m}{2} + \left(\frac{1-k_1}{2}\right) Y_h Z_m + \left(\frac{1-k_1}{4}\right) Y_h Y_m\right)/\overline{w}_h$$
$$\overline{w}_m = 1 - \frac{Y_h}{2}\left(X_m k_2 + Y_m\left(\frac{k_1+k_2}{2}\right) + Z_m k_1\right)$$

These equations can be consolidated to a system of equations for the four sex-specific allele frequencies *p* and heterozygosities *Y*:

$$p'_h = \left(S\left(p_h - \frac{Y_h k_2}{4}\right) + b(1-S)\left(\frac{p_h + p_m - Y_h k_2 p_m}{2}\right)\right)/\overline{w}_h$$
$$Y'_h = \left(S\frac{Y_h}{2} + b(1-S)(p_h q_m + q_h p_m)\right)/\overline{w}_h$$
$$\overline{w}_h = S\left(1 - \frac{Y_h}{2}\left(\frac{k_1 + k_2}{2}\right)\right) + b(1-S)\left(1 - \frac{Y_h}{2}(p_m k_2 + q_m k_1)\right)$$
$$p'_m = \left(\frac{p_h + p_m - Y_h k_2 p_m}{2}\right)/\overline{w}_m$$
$$Y'_m = \frac{p_h q_m + q_h p_m}{\overline{w}_m}$$
$$\overline{w}_m = 1 - \frac{Y_h}{2}(p_m k_2 + q_m k_1)$$

These recursions can be rewritten to highlight the effects of the *Medea* alleles, as shown in Table S1:

$$p'_h = \left(p_h\left(S + \frac{b-Sb}{2}\right) + p_m\left(\frac{b-Sb}{2}\right) - \frac{Y_h}{2}k_2\left(\frac{S}{2} + bp_m - Sbp_m\right)\right)/\overline{w}_h$$
$$Y'_h = \left(S\frac{Y_h}{2} + b(1-S)(p_h q_m + q_h p_m)\right)/\overline{w}_h$$
$$\overline{w}_h = S + b - Sb - \frac{Y_h}{2}\left(k_1\left(\frac{S}{2} + bq_m - Sbq_m\right) + k_2\left(\frac{S}{2} + bp_m - Sbp_m\right)\right)$$
$$p'_m = \left(\frac{p_h + p_m - Y_h k_2 p_m}{2}\right)/\overline{w}_m$$
$$Y'_m = \frac{p_h q_m + q_h p_m}{\overline{w}_m}$$
$$\overline{w}_m = 1 - \frac{Y_h}{2}(p_m k_2 + q_m k_1)$$

Recursions for *peel* elements under *Caenorhabditis*-type androdioecy

The mating table below shows the effects of *peel* alleles (P_1 and P_2) in an androdioecious population.

Egg-parent	Sperm-parent	Frequency	Surviving Progeny Genotypes: Hermaphrodites P_1P_1	Hermaphrodites P_1P_2	Hermaphrodites P_2P_2	Males P_1P_1	Males P_1P_2	Males P_2P_2
	P_1P_1	SX_h	1					
	P_1P_2	SY_h	$(1-k_2)/4$	1/2	$(1-k_1)/4$			
	P_2P_2	SZ_h			1			
P_1P_1	P_1P_1	$(1-S)X_hX_P$	b			b		
P_1P_1	P_1P_2	$(1-S)X_hY_P$	$b(1-k_2)/2$	$b/2$		$b(1-k_2)/2$	$b/2$	
P_1P_1	P_2P_2	$(1-S)X_hZ_P$		b			b	
P_1P_2	P_1P_1	$(1-S)Y_hX_P$	$b/2$	$b/2$		$b/2$	$b/2$	
P_1P_2	P_1P_2	$(1-S)Y_hY_P$	$b(1-k_2)/4$	$b/2$	$b(1-k_1)/4$	$b(1-k_2)/4$	$b/2$	$b(1-k_1)/4$
P_1P_2	P_2P_2	$(1-S)Y_hZ_P$		$b/2$	$b/2$		$b/2$	$b/2$
P_2P_2	P_1P_1	$(1-S)Z_hX_P$		b			b	
P_2P_2	P_1P_2	$(1-S)Z_hY_P$		$b/2$	$b(1-k_1)/2$		$b/2$	$b(1-k_1)/2$
P_2P_2	P_2P_2	$(1-S)Z_hZ_P$			b			b

This table yields the following recursions for genotype frequencies:

$$X'_h = (S\left(X_h + \left(\frac{1-k_2}{4}\right)Y_h\right) + b(1-S)\left(X_hX_m + \frac{Y_hX_m}{2} + \left(\frac{1-k_2}{2}\right)X_hY_m + \left(\frac{1-k_2}{4}\right)Y_hY_m\right))/\overline{w}_h$$

$$Y'_h = (S\frac{Y_h}{2} + b(1-S)\left(X_hZ_m + \frac{Y_h + Y_m - Y_hY_m}{2} + Z_hX_m\right))/\overline{w}_h$$

$$Z'_h = (S\left(Z_h + \left(\frac{1-k_1}{4}\right)Y_h\right) + b(1-S)\left(Z_hZ_m + \frac{Y_hZ_m}{2} + \left(\frac{1-k_1}{2}\right)Z_hY_m + \left(\frac{1-k_1}{4}\right)Y_hY_m\right))/\overline{w}_h$$

$$\overline{w}_h = S\left(1 - Y_h\left(\frac{k_1 + k_2}{4}\right)\right) + b(1-S)\left(1 - \frac{Y_m}{2}\left(X_hk_2 + Y_h\left(\frac{k_1+k_2}{2}\right) + Z_hk_1\right)\right)$$

$$X'_m = \left(X_hX_m + \frac{Y_hX_m}{2} + \left(\frac{1-k_2}{2}\right)X_hY_m + \left(\frac{1-k_2}{4}\right)Y_hY_m\right))/\overline{w}_m$$

$$Y'_m = \left(X_hZ_m + \frac{Y_h + Y_m - Y_hY_m}{2} + Z_hX_m\right)/\overline{w}_m$$

$$Z'_m = \left(Z_hZ_m + \frac{Y_hZ_m}{2} + \left(\frac{1-k_1}{2}\right)Z_hY_m + \left(\frac{1-k_1}{4}\right)Y_hY_m\right)/\overline{w}_h$$

$$\overline{w}_m = 1 - \frac{Y_m}{2}(X_hk_2 + Y_h\left(\frac{k_1+k_2}{2}\right) + Z_hk_1)$$

These equations can be consolidated to a system of equations for the four sex-specific allele frequencies p and heterozygosities Y:

$$p'_h = \left(S\left(p_h - \frac{Y_hk_2}{4}\right) + b(1-S)\left(\frac{p_h + p_m - Y_mk_2p_h}{2}\right)\right)/\overline{w}_h$$

$$Y'_h = \left(S\frac{Y_h}{2} + b(1-S)(p_hq_m + q_hp_m)\right)/\overline{w}_h$$

$$\overline{w}_h = S\left(1 - \frac{Y_h}{2}\left(\frac{k_1 + k_2}{2}\right)\right) + b(1-S)\left(1 - \frac{Y_m}{2}(p_hk_2 + q_hk_1)\right)$$

$$p'_m = \left(\frac{p_h + p_m - Y_m k_2 p_h}{2}\right) / \overline{w}_m$$
$$Y'_m = \frac{p_h q_m + q_h p_m}{\overline{w}_m}$$
$$\overline{w}_m = 1 - \frac{Y_m}{2}(p_h k_2 + q_h k_1)$$

And these can be rewritten to highlight the effects of the *peel* alleles, as shown in Table S1:

$$p'_h = \left(p_h\left(S + \frac{b - Sb}{2}\right) + p_m\left(\frac{b - Sb}{2}\right) - \frac{k_2}{2}\left(Y_h\frac{S}{2} + Y_m(bp_h - Sbp_h)\right)\right) / \overline{w}_h$$
$$Y'_h = \left(S\frac{Y_h}{2} + b(1 - S)(p_h q_m + q_h p_m)\right) / \overline{w}_h$$
$$\overline{w}_h = S + b - Sb - \left(\frac{k_1}{2}\left(Y_h\frac{S}{2} + Y_m(bq_h - Sbq_h)\right) + \frac{k_2}{2}\left(Y_h\frac{S}{2} + Y_m(bp_h - Sbp_h)\right)\right)$$
$$p'_m = \left(\frac{p_h + p_m}{2} - \frac{Y_m k_2 p_h}{2}\right) / \overline{w}_m$$
$$Y'_m = \frac{p_h q_m + q_h p_m}{\overline{w}_m}$$
$$\overline{w}_m = 1 - \frac{Y_m}{2}(p_h k_2 + q_h k_1)$$

Recursions for *Medea*-peel antagonism under *Caenorhabditis*-type androdioecy

The table below represents the case of antagonistic *Medea* and *peel* alleles (*M* and *P*) in an androdioecious population, where the penetrances of *Medea* and *peel* are k_M and k_P respectively. Note that this model is not symmetrical the way the other models are. Here we let *X, Y,* and *Z* be the genotype frequencies of *Medea* homozygotes, heterozygotes, and *peel* homozygotes, respectively. Consequently, *p* is the *Medea* allele frequency and 1-*p* = *q* is the *peel* allele frequency.

			Surviving Progeny Genotypes					
Herm	Male	Frequency	Hermaphrodites			Males		
			M_1M_1	M_1P_2	P_2P_2	M_1M_1	M_1P_2	P_2P_2
MM		SX_h	1					
MP		SY_h	$(1-k_P)/4$	1/2	$(1-k_M)/4$			
PP		SZ_h			1			
MM	*MM*	$(1-S)X_hX_m$	b			b		
MM	*MP*	$(1-S)X_hY_m$	$b(1-k_P)/2$	$b/2$		$b(1-k_P)/2$	$b/2$	
MM	*PP*	$(1-S)X_hZ_m$		b			b	
MP	*MM*	$(1-S)Y_hX_m$	$b/2$	$b/2$		$b/2$	$b/2$	
MP	*MP*	$(1-S)Y_hY_m$	$b(1-k_P)/4$	$b/2$	$b(1-k_M)/4$	$b(1-k_P)/4$	$b/2$	$b(1-k_M)/4$
MP	*PP*	$(1-S)Y_hZ_m$		$b/2$	$b(1-k_M)/2$		$b/2$	$b(1-k_M)/2$
PP	*MM*	$(1-S)Z_hX_m$		b			b	
PP	*MP*	$(1-S)Z_hY_m$		$b/2$	$b/2$		$b/2$	$b/2$
PP	*PP*	$(1-S)Z_hZ_m$			b			b

This table yields the following recursions for genotype frequencies:

$$X'_h = \left(S\left(X_h + \left(\frac{1-k_P}{4}\right)Y_h\right) + b(1-S)\left(X_hX_m + \frac{Y_hX_m}{2} + \left(\frac{1-k_P}{2}\right)X_hY_m + \left(\frac{1-k_P}{4}\right)Y_hY_m\right)\right)/\bar{w}_h$$

$$Y'_h = \left(S\frac{Y_h}{2} + b(1-S)\left(X_hZ_m + \frac{Y_h+Y_m-Y_hY_m}{2} + Z_hX_m\right)\right)/\bar{w}_h$$

$$Z'_h = \left(S\left(Z_h + \left(\frac{1-k_M}{4}\right)Y_h\right) + b(1-S)\left(Z_hZ_m + \frac{Z_hY_m}{2} + \left(\frac{1-k_M}{2}\right)Y_hZ_m + \left(\frac{1-k_M}{4}\right)Y_hY_m\right)\right)/\bar{w}_h$$

$$\bar{w}_h = S\left(1 - Y_h\left(\frac{k_M + k_P}{4}\right)\right) + b(1-S)\left(1 - \frac{Y_mk_P}{2}\left(X_h + \frac{Y_h}{2}\right) - \frac{Y_hk_M}{2}\left(Z_m + \frac{Y_m}{2}\right)\right)$$

$$X'_m = \left(X_hX_m + \frac{Y_hX_m}{2} + \left(\frac{1-k_P}{2}\right)X_hY_m + \left(\frac{1-k_P}{4}\right)Y_hY_m\right)/\bar{w}_m$$

$$Y'_m = \left(X_hZ_m + \frac{Y_h+Y_m-Y_hY_m}{2} + Z_hX_m\right)/\bar{w}_m$$

$$Z'_m = \left(Z_hZ_m + \frac{Z_hY_m}{2} + \left(\frac{1-k_M}{2}\right)Y_hZ_m + \left(\frac{1-k_M}{4}\right)Y_hY_m\right)/\bar{w}_m$$

$$\bar{w}_m = 1 - \frac{Y_mk_P}{2}\left(X_h + \frac{Y_h}{2}\right) - \frac{Y_hk_M}{2}\left(Z_m + \frac{Y_m}{2}\right)$$

These equations can be consolidated to a system of equations for the four sex-specific allele frequencies *p* and heterozygosities *Y*:

$$p'_h = \left(S\left(p_h - \frac{Y_h k_P}{4}\right) + b(1-S)\left(\frac{p_h + p_m - Y_m k_P p_h}{2}\right)\right)/\bar{w}_h$$
$$Y'_h = \left(S\frac{Y_h}{2} + \ b(1-S)(p_h q_m + q_h p_m)\right)/\bar{w}_h$$
$$\bar{w}_h = S\left(1 - \frac{Y_h}{2}\left(\frac{k_P + \ k_M}{2}\right)\right) + b(1-S)\left(1 - (\frac{Y_m p_h k_P + Y_h q_m k_M}{2})\right)$$
$$p'_m = \left(\frac{p_h + p_m - Y_m k_P p_h}{2}\right)/\bar{w}_m$$
$$Y'_m = \frac{p_h q_m + q_h p_m}{\bar{w}_m}$$
$$\bar{w}_m = 1 - \frac{1}{2}(Y_m k_P p_h + Y_h k_M q_m)$$

And these recursions can be rewritten to highlight the effects of the *Medea* and *peel* alleles, as shown in Table S1:

$$p'_h = \left(p_h\left(S + \frac{b - Sb}{2}\right) + p_m\left(\frac{b - Sb}{2}\right) - \frac{k_P}{2}\left(Y_h\frac{S}{2} + Y_m(bp_h - Sbp_h)\right)\right)/\bar{w}_h$$
$$Y'_h = \left(S\frac{Y_h}{2} + \ b(1-S)(p_h q_m + q_h p_m)\right)/\bar{w}_h$$
$$\bar{w}_h = S + b - Sb - \left(\frac{k_P}{2}\left(Y_h\frac{S}{2} + Y_m(bp_h - Sbp_h)\right) + \frac{k_M}{2}Y_h\left(\frac{S}{2} + (bq_m - Sbq_m)\right)\right)$$
$$p'_m = \left(\frac{p_h + p_m}{2} - \frac{Y_m k_P p_h}{2}\right)/\bar{w}_m$$
$$Y'_m = \frac{p_h q_m + q_h p_m}{\bar{w}_m}$$
$$\bar{w}_m = 1 - \frac{1}{2}(Y_m k_P p_h + Y_h k_M q_m)$$

Monoecy	Androdioecy, *Medea*	Androdioecy, *peel*	Androdioecy, *Medea* & *peel*
$p' = \frac{\left(p - \frac{Y}{2}k_2\left(\frac{S}{2} + p - Sp\right)\right)}{\bar{w}_h}$<br>$Y' = (S\frac{Y}{2} + (1-S)2pq)/\bar{w}$<br>$\bar{w} = 1 - \frac{Y}{2}\left(k_1\left(\frac{S}{2} + q - Sq\right) + k_2\left(\frac{S}{2} + p - Sp\right)\right)$	$p'_h = \frac{p_h\left(S + \frac{b-Sb}{2}\right) + p_m\left(\frac{b-Sb}{2}\right) - \frac{Y_h}{2}k_2\left(\frac{S}{2} + bp_m - Sbp_m\right)}{\bar{w}_h}$<br>$Y'_h = \left(S\frac{Y_h}{2} + b(1-S)(p_h q_m + q_h p_m)\right)/\bar{w}_h$<br>$\bar{w}_h = S + b - Sb - \frac{Y_h}{2}\left(k_1\left(\frac{S}{2} + bq_m - Sbq_m\right) + k_2\left(\frac{S}{2} + bp_m - Sbp_m\right)\right)$	$p'_h = \frac{p_h\left(S + \frac{b-Sb}{2}\right) + p_m\left(\frac{b-Sb}{2}\right) - \frac{k_2}{2}\left(Y_h\frac{S}{2} + Y_m(bp_h - Sbp_h)\right)}{\bar{w}_h}$<br>$Y'_h = \left(S\frac{Y_h}{2} + b(1-S)(p_h q_m + q_h p_m)\right)/\bar{w}_h$<br>$\bar{w}_h = S + b - Sb - \left(\frac{k_1}{2}\left(Y_h\frac{S}{2} + Y_m(bq_h - Sbq_h)\right) + \frac{k_2}{2}\left(Y_h\frac{S}{2} + Y_m(bp_h - Sbp_h)\right)\right)$	$p'_h = \frac{p_h\left(S + \frac{b-Sb}{2}\right) + p_m\left(\frac{b-Sb}{2}\right) - \frac{k_P}{2}\left(Y_h\frac{S}{2} + Y_m(bp_h - Sbp_h)\right)}{\bar{w}_h}$<br>$Y'_h = \left(S\frac{Y_h}{2} + b(1-S)(p_h q_m + q_h p_m)\right)/\bar{w}_h$<br>$\bar{w}_h = S + b - Sb - \left(\frac{k_P}{2}\left(Y_h\frac{S}{2} + Y_m(bp_h - Sbp_h)\right) + \frac{k_M}{2}Y_h\left(\frac{S}{2} + (bq_m - Sbq_m)\right)\right)$
Monoecy or Dioecy, obligate outcrossing ($S = 0$)			
$p' = (p - \frac{Y}{2}k_2 p)/\bar{w}$<br>$Y' = 2pq/\bar{w}$<br>$\bar{w} = 1 - \frac{Y}{2}(k_1 q + k_2 p)$	$p'_m = \left(\frac{p_h + p_m}{2} - \frac{Y_h}{2}k_2 p_m\right)/\bar{w}_m$<br>$Y'_m = (p_h q_m + q_h p_m)/\bar{w}_m$<br>$\bar{w}_m = 1 - \frac{Y_h}{2}(k_1 q_m + k_2 p_m)$	$p'_m = \left(\frac{p_h + p_m}{2} - \frac{Y_m}{2}k_2 p_h\right)/\bar{w}_m$<br>$Y'_m = (p_h q_m + q_h p_m)/\bar{w}_m$<br>$\bar{w}_m = 1 - \frac{Y_m}{2}(k_1 q_h + k_2 p_h)$	$p'_m = \left(\frac{p_h + p_m}{2} - \frac{Y_m}{2}k_P p_h\right)/\bar{w}_m$<br>$Y'_m = (p_h q_m + q_h p_m)/\bar{w}_m$<br>$\bar{w}_m = 1 - \left(\frac{Y_m}{2}k_P p_h + \frac{Y_h}{2}k_M q_m\right)$

Table S1. Comparison of recursion equations for different mating systems.
Sex-specific recursion equations under androdioecy resemble the monoecious case for hermaphrodites and the obligately outcrossing case for males. Important differences are that the allele frequency p is replaced by its sex-weighted average in hermaphrodites and its unweighted average in males, and the generation of heterozygotes by outcrossing, $2pq$ under monoecy or dioecy, is $p_h q_m + q_h p_m$ under androdioecy, influenced by sex differences in allele frequencies. Finally, *Medea* and *peel* elements differ in which sex's heterozygosity and allele frequency influence the effects of the alleles.

Variables and parameters:

p	M_1 allele frequency
q	M_2 allele frequency, 1-p
Y	M_1M_2 heterozygote genotype frequency
$\bar{w}$	Population mean fitness, the proportion of zygotes that survive to reproduce
S	Selfing rate
b	Ratio of hermaphrodite progeny from an outcrossing to hermaphrodite progeny from a selfing
k_1	penetrance of the M_1 allele
k_2	penetrance of the M_2 allele
k_M	penetrance of the *Medea* allele in the *Medea-peel* antagonism model
k_P	penetrance of the *peel* allele in the *Medea-peel* antagonism model
h	subscript for hermaphrodite-specific variables
m	subscript for male-specific variables

Figure S3 (caption on next page)

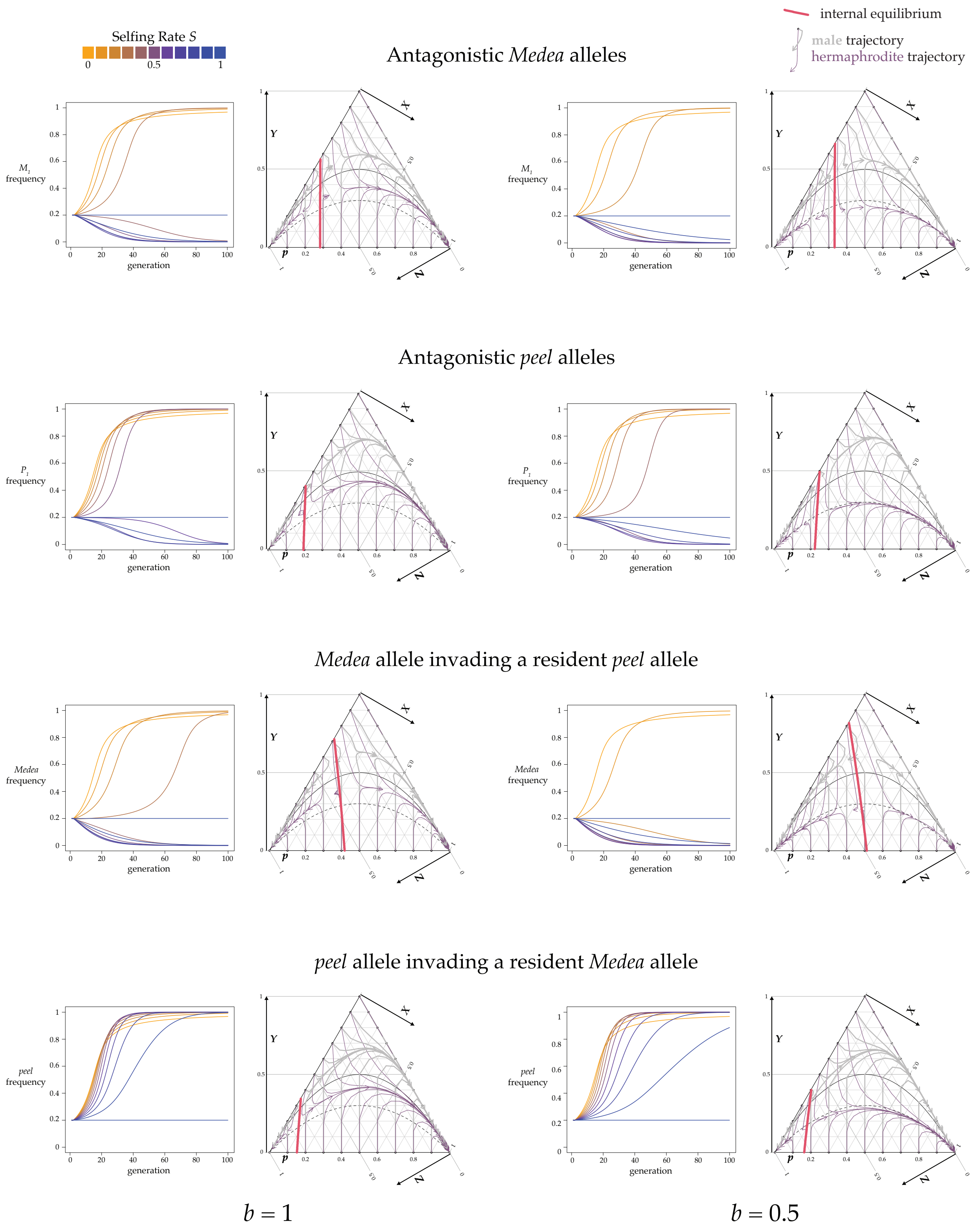

Figure S3. The effects of *Caenorhabditis*-type androdioecy on genotype and allele-frequency evolution differ between *Medea* and *peel* alleles, and are driven by large differences in heterozygosity between males and hermaphrodites. The four rows show the dynamics for antagonistic *Medeas*, antagonistic peels, a *Medea* invading a population with a resident *peel*, and a *peel* invading a population with a resident *Medea*. The left half of the figure shows results for $b = 1$, and the right half $b = 0.5$. For each of the eight situations, there are two plots. The left one shows allele frequencies found by iterating the relevant equations (Supplementary File 1) for eleven values of S from 0 to 1, with initial M_1 frequency $p = 0.2$ and heterozygosity $Y = 0$. The resident *Medea* M_2 has penetrance $k_2 = 0.65$ and the invading *Medea* M_1 has penetrance $k_1 = 1$. The right panel in each pair shows the dynamics in genotype space via De Finetti plot. These examples show again the case of $k_1 = 1$ and $k_2 = 0.65$, here with fixed selfing rate $S = 0.57$. Each trajectory starts from one position at the periphery of the plot and represents the male- or hermaphrodite-specific genotype frequencies through 15 generations. The red line shows the unstable internal equilibrium, conditional on identical starting frequencies for males and females. Under androdioecy, the equilibrium is sensitive to the initial heterozygosity (as seen in its departures from the vertical). To the right of this line, the invading allele fixes, and to the left, it is eliminated. The solid black curve shows genotype frequencies at Hardy-Weinberg equilibrium, and the dashed curve shows genotype frequencies under the neutral extended Hardy Weinberg equilibrium with selfing.

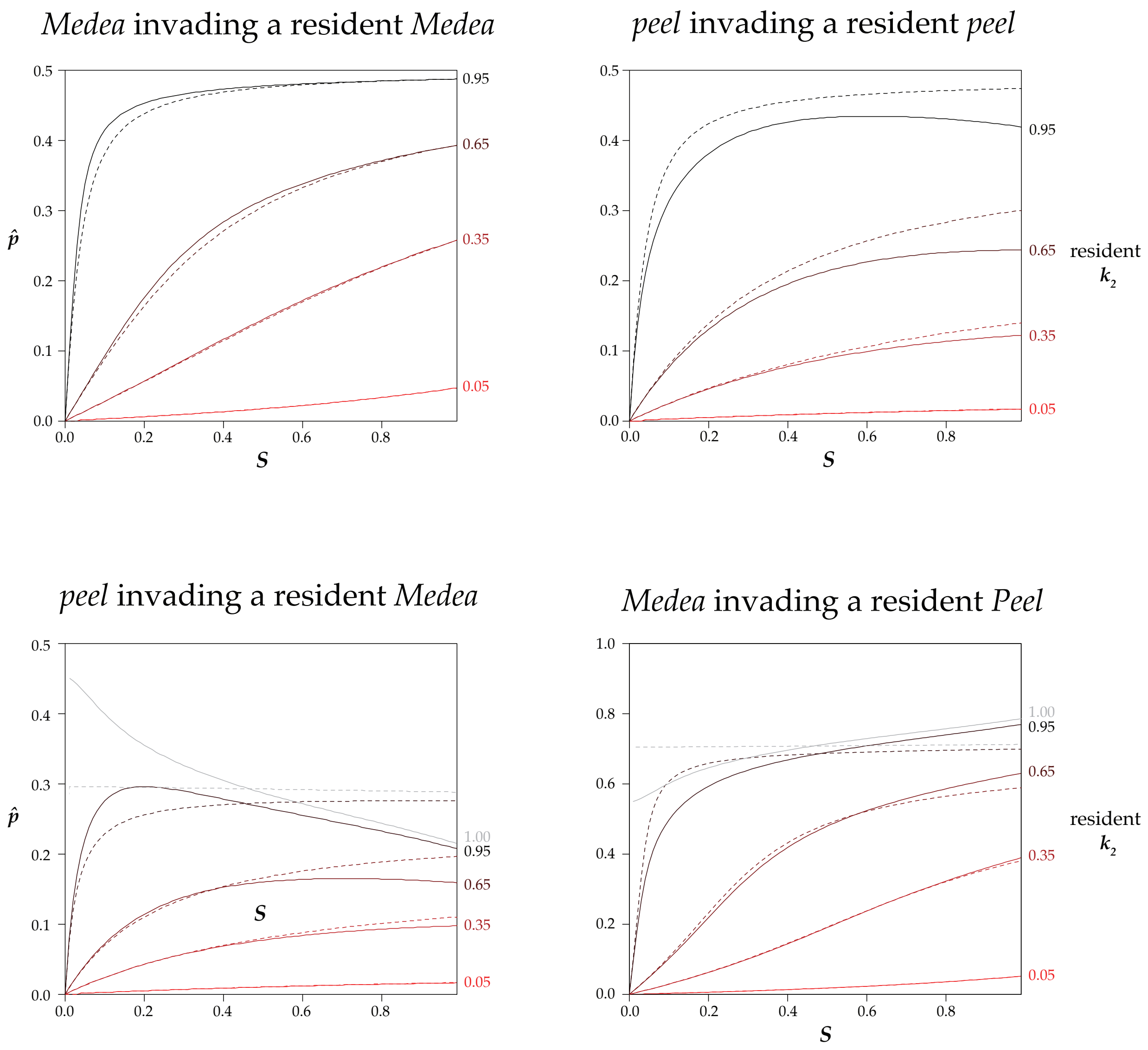

Figure S4. Frequency thresholds for invasion, with b = 0.5. This figure differs from Figure 5 only in the value of b. In an androdioecious population with a resident allele with the specified penetrance (k_2), a completely penetrant allele (k_1=1) can invade and sweep to fixation if its frequency p is above the relevant thick solid line at the indicated selfing rate (S). Below the line, the resident excludes the invader. The dashed lines describe the results for an androdioecious population where the k values are ten-fold lower. For example, the topmost dashed line represents the case of k_2 = 0.095 and k_1 = 0.1.
