# Supplementary material for: Parental-effect gene-drive elements under partial selfing, or why do Caenorhabditis genomes have hyperdivergent regions?
Source: Genetics. 2024 Oct 30;229(1):iyae175. doi: 10.1093/genetics/iyae175 (PMC11708918; doi:10.1093/genetics/iyae175)
Supplement: iyae175_Supplementary_Data [file iyae175_supplementary_data.zip › Supplemental_Material_Legends_GENETICS-2024-307305.docx]

**SUPPLEMENTARY FILES**

**File S1. Supplementary text**

**Pages Content**

1 Special Case 1: A single *Medea,* with Figure S1.

2-3 Special Case 2: Two *Medeas* with equal penetrance, with Figure S2.

4-9 Recursion equations under androdioecy

10 Table S1. Comparison of recursion equations for different mating systems.

11-12 Figure S3.

13 Figure S4

**File S2: R package** *MedeaFight*.

This zipped directory also contains an html guide to using the package and an R script, *PlotMedeaFigures*, that allows for reconstruction of the figures from the manuscript. To use the package, download and unzip the file and then in R, install.packages(“<path to directory> /MedeaFightAndGuide/MedeaFight”, repos = NULL, type = “source”). Finally, library(“MedeaFight”). For some functions it is necessary to also have the Ternary package, which is available from CRAN, at cran.r-project.org.
